# Structure-Based Identification and Biological Characterization of New NAPRT Inhibitors

**DOI:** 10.3390/ph15070855

**Published:** 2022-07-12

**Authors:** Jorge Franco, Francesco Piacente, Melanie Walter, Simone Fratta, Moustafa Ghanem, Andrea Benzi, Irene Caffa, Alexander V. Kurkin, Andrea Altieri, Patrick Herr, Macarena Martínez-Bailén, Inmaculada Robina, Santina Bruzzone, Alessio Nencioni, Alberto Del Rio

**Affiliations:** 1 Innovamol Consulting Srl, 41126 Modena, Italy; jorfrasus1995@gmail.com; 2 Department of Internal Medicine and Medical Specialties, University of Genoa, 16132 Genoa, Italy; moustafa.ghanem@edu.unige.it (M.G.); irene.caffa@libero.it (I.C.); alessio.nencioni@unige.it (A.N.); 3 Department of Experimental Medicine, University of Genoa, 16132 Genoa, Italy; francesco.piacente@unige.it (F.P.); andreeabenzi@gmail.com (A.B.); santina.bruzzone@unige.it (S.B.); 4 Department of Oncology and Metabolism, University of Sheffield, Sheffield S10 2TN, UK; mwalter3@sheffield.ac.uk (M.W.); p.herr@sheffield.ac.uk (P.H.); 5 Department of Organic Chemistry, University of Seville, 41012 Seville, Spain; sfratta@us.es (S.F.); mmartinez45@us.es (M.M.-B.); robina@us.es (I.R.); 6 Department of Chemistry, Lomonosov Moscow State University, 119991 Moscow, Russia; indolester@gmail.com (A.V.K.); aaltieri@edasascientific.com (A.A.); 7 EDASA Scientific Srls, 66050 San Salvo, Italy; 8 IRCCS Ospedale Policlinico San Martino, 16132 Genoa, Italy; 9 Institute of Organic Synthesis and Photoreactivity (ISOF), National Research Council (CNR), 40129 Bologna, Italy

**Keywords:** NAPRT inhibitors, NAD biosynthesis, Preiss–Handler pathway, OVCAR-5, bioactive molecules, NAMPT, molecular design, virtual screening

## Abstract

NAPRT, the rate-limiting enzyme of the Preiss–Handler NAD biosynthetic pathway, has emerged as a key biomarker for the clinical success of NAMPT inhibitors in cancer treatment. Previous studies found that high protein levels of NAPRT conferred resistance to NAMPT inhibition in several tumor types whereas the simultaneous blockade of NAMPT and NAPRT results in marked anti-tumor effects. While research has mainly focused on NAMPT inhibitors, the few available NAPRT inhibitors (NAPRTi) have a low affinity for the enzyme and have been scarcely characterized. In this work, a collection of diverse compounds was screened in silico against the NAPRT structure, and the selected hits were tested through cell-based assays in the NAPRT-proficient OVCAR-5 ovarian cell line and on the recombinant hNAPRT. We found different chemotypes that efficiently inhibit the enzyme in the micromolar range concentration and for which direct engagement with the target was verified by differential scanning fluorimetry. Of note, the therapeutic potential of these compounds was evidenced by a synergistic interaction between the NAMPT inhibitor FK866 and the new NAPRTi in terms of decreasing OVCAR-5 intracellular NAD levels and cell viability. For example, compound IM29 can potentiate the effect of FK866 of more than two-fold in reducing intracellular NAD levels. These results pave the way for the development of a new generation of human NAPRTi with anticancer activity.

## 1. Introduction

Nicotinamide adenine dinucleotide (NAD) is a vital pyridine nucleotide. The first role that was discovered for NAD^+^ and its phosphorylated form (NADP^+^) was as an essential coenzyme in redox reactions that are involved in cell energy and anabolic metabolism. By exchanging hydride, NAD(P)^+^ is constantly shuttling between its oxidized and reduced forms in hundreds of enzymatic reactions that take part in key pathways in mammalian cells, such as glycolysis, tricarboxylic acid cycle (TCA cycle), oxidative phosphorylation, and serine biosynthesis [1,2,3]. In addition, marked cell regulatory properties have been ascribed to NAD by acting as a substrate for several families of enzymes, which always release nicotinamide (Nam) as a result of NAD degradation [1,4,5,6]. Indeed, NAD is consumed in post-translational modifications of target proteins by mono- and poly-(ADP–ribose) polymerases (PARPs) and sirtuins (SIRT1-7), the last endowed with protein deac(et)ylase activity [7,8]. NAD is also the precursor of the Ca^2+^-mobilizing second messenger cyclic ADP-ribose (cADPR), produced by the ectoenzymes CD38 and CD157 [9]. The enzyme sterile alpha and TIR motif-containing 1 (SARM1) exerts NAD-cleavage activity in neurons and represents a new family of NAD-consuming enzymes [10]. The activities of these enzymes are modulated by NAD availability and regulate a series of fundamental cellular processes including DNA repair, apoptosis, cell metabolism, cell cycle progression, and immune responses [11].

Unlike a redox cofactor, NAD is consumed when acting as a substrate. Therefore, continuous NAD biosynthesis is required in normal human tissues to preserve NAD homeostasis and thus health [12]. Due to aberrant metabolism, cell growth, and proliferation, tumor cells require higher NAD production with respect to healthy tissues to support the increased activity of NAD-degrading enzymes [13]. Therefore, interfering with the NAD biosynthetic machinery was conceived as a promising therapeutic strategy against cancer [11,14]. The depletion of NAD strongly affects multiple cellular metabolic pathways, leads to a rapid decline in adenosine triphosphate (ATP) levels, and ultimately causes cancer cell death [15]. For a more detailed overview of the in vitro and in vivo effects of chemical agents targeting NAD biosynthesis in cancer cells, we refer the readers to recent review articles [16].

**Figure 1:**
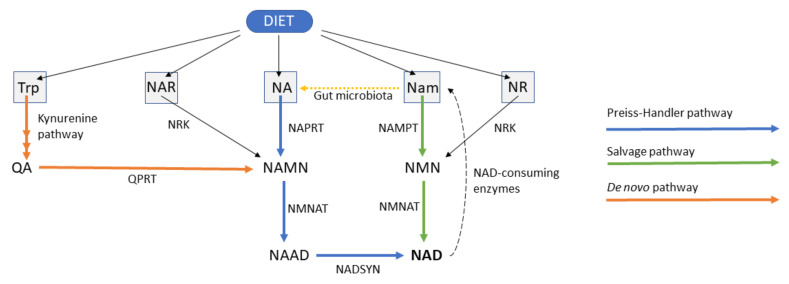
Graphical depiction of NAD biosynthesis in mammals. Trp, tryptophan; NA, nicotinic acid; NAR, nicotinic acid riboside; NR, nicotinamide riboside; Nam, nicotinamide; QA, quinolinic acid; NAMN, nicotinic acid mononucleotide; NMN, nicotinamide mononucleotide; NAAD, nicotinic acid adenine dinucleotide; NAD, nicotinamide adenine dinucleotide; QPRT, quinolinate phosphoribosyltransferase; NAPRT, nicotinate phosphoribosyltransferase; NAMPT, nicotinamide phosphoribosyltransferase; NRK, nicotinamide riboside kinase; NMNAT, nicotinamide mononucleotide adenylyltransferase; NADSYN, nicotinamide adenine dinucleotide synthetase.

There are three main pathways contributing to NAD biosynthesis in mammals: the de novo pathway with tryptophan as a NAD precursor, the Preiss–Handler pathway, which utilizes nicotinic acid (NA) as a starting block, and the nicotinamide (Nam) salvage pathway (Figure 1) [17]. In addition, the ribosylated precursors nicotinamide riboside (NR) and nicotinic acid riboside (NAR) represent additional forms of vitamin B3 that can be converted into NAD.

The Nam salvage pathway plays a key role in maintaining NAD homeostasis in mammalian cells [18]. Duarte-Pereira and colleagues observed that the gene of the rate-limiting enzyme of this pathway, nicotinamide phosphoribosyltransferase (NAMPT), was ubiquitously expressed at the mRNA level in all human normal tissues and tumors that were studied [19]. Moreover, NAD-consuming enzymes release nicotinamide as a by-product, which makes Nam the most accessible NAD precursor. Accordingly, research focusing on NAD-lowering as an anticancer strategy has largely focused on NAMPT and led to the identification of numerous inhibitors, including FK866 and CHS-828 [20,21,22]. Despite the potent antitumor activity of these agents in preclinical models, the efficacy of these compounds in clinical trials has been disappointing. In addition, thrombocytopenia and gastrointestinal symptoms arose as dose-limiting toxicities [23,24]. Studies show that the limited clinical activity of these agents reflects the overexpression of enzymes from the Preiss–Handler pathway, at least in a subset of human malignancies [25,26].

Human nicotinate phosphoribosyltransferase (hNAPRT, Uniprot: Q6XQN6) is the rate-limiting enzyme of the Preiss–Handler pathway. The enzyme catalyzes the conversion of NA and 5-phosphoribosyl-1-pyrophosphate (PRPP) to nicotinic acid mononucleotide (NAMN) and pyrophosphate (PPi) in an ATP-dependent manner [27]. The Preiss–Handler pathway continues with the adenylation of NAMN catalyzed by NMNAT1-3 and ends with the amidation of NAAD into NAD, which is catalyzed by NAD synthetase (NADSYN). Human NAPRT belongs to the Type II phosphoribosyltransferase family of functional dimeric proteins that are involved in NAD biosynthesis, together with quinolinate phosphoribosyltransferase (QPRT) and NAMPT. The crystal structure of hNAPRT was solved by Marletta et al. and evidenced that the NAPRT monomer consists of an irregular α/β barrel domain and of a second open-faced sandwich domain [28]. Despite the low sequence similarity between hNAPRT and its bacterial homologs, the main amino acids that are involved in the recognition and stabilization of NAPRT substrates are strictly conserved, as demonstrated through site-directed mutagenesis experiments [29].

NAPRT displays marked tissue and tumor specificity in terms of expression and its regulation mechanisms and is mostly present in several catabolic healthy mammalian tissues including the heart, kidney, liver, and small intestine [19,25,30,31,32,33]. In tissues that express the NAPRT protein, NA is the preferred precursor of NAD. Accordingly, the NAD pool of HEK293 cells was dramatically increased when these were cultured in NA-rich conditions, whereas similar doses of Nam resulted in a much lower effect [33]. This observation is likely related to the fact that, unlike NAMPT, NAPRT activity is not inhibited by NAD [33]. Interestingly, in addition to the role of NAMPT and NAPRT as intracellular NAD-producing enzymes (mostly located in the nucleus and the cytoplasm), NAMPT and NAPRT also exist as extracellular proteins, which exert pro-inflammatory and pro-tumorigenic effects [34,35].

With respect to the amplification of the NAPRT gene and to its expression, these are very variable in human tumors [19,25,31]. Tumors originating from normal tissues that highly express NAPRT were found to amplify the NAPRT gene at a high frequency (and to express it at high levels as a result), whereas tumors arising from tissues that do not express NAPRT will strongly rely on NAMPT activity for NAD biosynthesis and for cell survival [31]. In ovarian, prostate, breast, and pancreatic cancers, NAPRT was to be found upregulated [25]. On the contrary, gastric, renal, and colorectal carcinoma, as well as several leukemia cell lines, were reported to have low or no NAPRT expression [19]. Among the mechanisms that regulate NAPRT expression, hypermethylation of the NAPRT gene promoter leads to gene silencing and has been found in several NAPRT-negative tumors. NAPRT promoter hypermethylation in cancer is frequently associated with mutations in the protein phosphatase Mg^2+^/Mn^2+^-dependent 1D (PPM1D) or isocitrate dehydrogenase 1 (IDH1) genes, as well as with the epithelial-mesenchymal transition (EMT)-subtype of gastric cancer [36,37,38]. Due to their dependency on the Nam salvage pathway for survival, NAPRT-deficient cancers are extremely sensitive to treatment with NAMPT inhibitors [39,40]. On the other hand, NAPRT upregulation confers resistance to NAMPT inhibitors to many malignancies. Piacente et al. demonstrated that NAPRT-proficient ovarian and pancreatic cancers are resistant to FK866, whereas NAPRT downregulation through gene silencing or chemical inhibition with 2-hydroxynicotinic acid (2-HNA) sensitized tumor cells to NAMPT inhibitors both in vitro and in vivo [25]. In line with these results, another study highlighted that sensitivity to NAMPT inhibition in several ovarian cancer cell lines was inversely proportional to NAPRT expression [41]. Despite its usefulness in basic research as NAPRTi, the low molecular weight and high functionality of 2-hydroxynicotinic acid suggest that this compound could have non-specific effects. Together with its low potency, such features make it unlikely that 2-HNA could be utilized as a NAPRTi in the clinic.

To the best of our knowledge, additional NAPRT inhibitors were reported in the 1970s, including nicotinic acid analogs and non-steroidal anti-inflammatory agents such as flufenamic acid, salicylic acid, mefenamic acid, and phenylbutazone [42,43,44]. Unfortunately, the available data, which were mostly obtained by monitoring NAPRT activity in human platelet lysates, suggest a weak inhibitory activity for these agents. Thus, the expected low specificity of 2-HNA combined with the limited activity and characterization of the other reported NAPRTi mandate the search for new, potent NAPRTi. In this work, we identified several new small-molecule NAPRTi which, upon further compound optimization, could lead to a new generation of molecules that sensitize NAPRT-proficient malignancies to NAMPT inhibitors and, possibly, to other anticancer agents.

## 2. Results

### 2.1. Sensitization of OVCAR-5 Cells to FK866

As described in the experimental section, a structure-based virtual screening was run on NAPRT to identify putative inhibitors. The docking results were thoroughly inspected and resulted in a selection of 62 compounds (Table 1 and Table S1 of Supplementary Information) to be tested employing in vitro assays on the ovarian cancer cell line OVCAR-5 that expresses high levels of NAPRT.

As noted in the methodology section, a 50 mM stock solution in DMSO was prepared for each compound. Compound IM 28 could not be solubilized in DMSO at the specified concentration and was excluded from the study.

**Figure 2:**
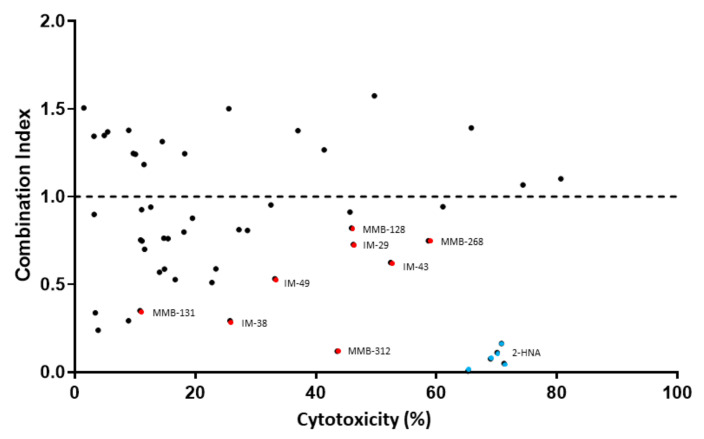
CI of compounds that were tested in combination with FK866 on OVCAR-5 cells. The cells were cultured in RPMI 1640 medium containing test compounds at a 100 μM concentration with and without 100 nM FK866 and the cell viability was determined following 72 h treatments. The data are shown as CI vs. cytotoxicity exerted on cells. Blue dots represent the reference NAPRT inhibitor, 2-HNA. Red dots belong to the best performing test compounds.

The first assay that we performed to test the putative NAPRT inhibitors was based on the principle that NAPRT-expressing OVCAR-5 cells are resistant to FK866 whereas the simultaneous chemical inhibition of NAPRT with 2-HNA renders the cells sensitive to the NAMPT inhibitor due to the cooperation between FK866 and 2-HNA in depleting the intracellular NAD pool [25]. We observed that, in line with the work that was conducted by Piacente et al., OVCAR-5 cells withstood 72 h treatments with 100 nM FK866 or 1 mM 2-HNA, whereas co-treatment with both compounds at the specified concentrations resulted in a marked synergistic effect exerting pronounced cell death (Figure 2). Thus, 2-HNA was used throughout the study as a positive control in our cell viability assay and any test compound resembling its activity was considered a potential NAPRT inhibitor. It is worth mentioning that, to reflect the physiological contribution of the Preiss–Handler pathway to NAD biosynthesis, all cell-based assays that are presented in this study were performed under a nicotinic acid concentration of 0.3 µM in the cell culture medium (Figure S1).

We decided to use combination index (CI) as a measure to determine the degree of interaction between FK866 and the test compounds i.e., to identify potential new NAPRT inhibitors. A CI < 1 indicates a synergistic interaction between the test compound and FK866 in decreasing cell viability. A CI = 1 and a CI > 1 are indicative of an additive and of an antagonistic effect, respectively.

Figure 2 shows that the simultaneous administration of FK866 and 2-HNA produced the lowest CI out of all the combinations that were tested (CI = 0.08) and marked cytotoxicity. Among all the tested compounds, eight (represented by red dots in Figure 2) were considered to be especially interesting for their low CI or their high cytotoxicity when they were combined with FK866. Compounds IM 29, IM 43, MMB-128, and MMB-268 exhibited a CI ranging between 0.62 and 0.82 and high cytotoxicity in combination with FK866 and these results might suggest other interactions within the cells while some degree of synergism (CI < 1) with FK866 was evident, suggesting the inhibition of the Preiss–Handler NAD biosynthetic pathway.

Compounds IM 38, IM 49, MMB-131, and MMB-312 produced less pronounced cytotoxicity when they were combined with FK866. However, this subset of compounds showed especially low combination indexes (0.12 ≤ CI ≤ 0.53) and, therefore, a larger synergism with FK866.

These eight compounds were all considered promising candidates for NAPRT inhibition and were selected for further in vitro assays.

### 2.2. Effect of Test Compounds on Intracellular NAD Levels

The assay that was performed on OVCAR-5 cell viability led to a set of potential NAPRT inhibitors, namely IM 29, IM 38, IM 43, IM 49, MMB-128, MMB-131, MMB-268, and MMB-312. In order to confirm that the cited compounds sensitized OVCAR-5 cells to the NAMPT inhibitor FK866 by interfering with NAD biosynthesis, we quantified the intracellular NAD^+^ levels in OVCAR-5 cells after 20-h treatments with these compounds with or without FK866.

**Figure 3:**
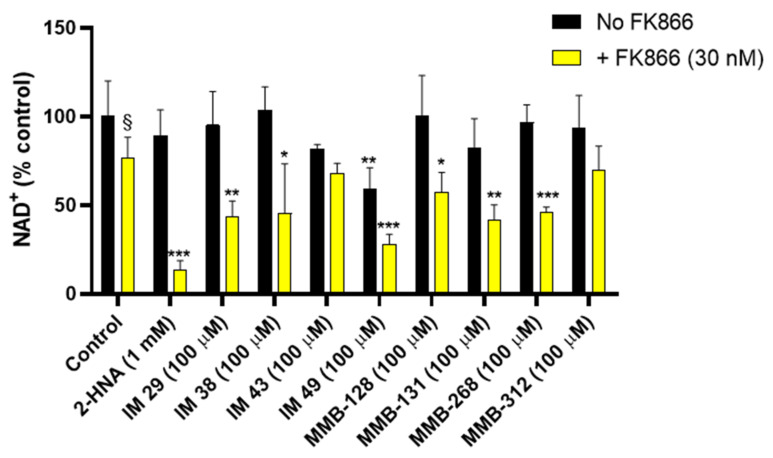
A subset of test compounds cooperates synergistically with FK866 in decreasing intracellular NAD^+^ levels in OVCAR-5 cells. The compounds were administered to cells alone or in combination with FK866 for 20 h. Quantification of NAD^+^ was performed via an enzyme cycling assay and normalized to cell lysate protein content. The results are the mean ± SD of two technical replicates with two biological replicates each. *, *p* < 0.05; **, *p* < 0.01; ***, *p* < 0.001 vs. the respective control, i.e., FK866-untreated control for the test compounds that were administered alone to cells, and FK866-treated control for the combinations of test compound and FK866; §, *p* < 0.05 versus FK866-untreated, control cells.

As Figure 3 shows, treatment with 30 nM FK866 alone decreased the NAD^+^ levels by 24%. The effect of FK866 was strongly enhanced when the drug was combined with 1 mM 2-HNA, yielding an 86 % drop in the intracellular NAD^+^ concentration, thus evidencing marked synergism between the two compounds in depleting NAD^+^. This observation is in line with the fact that the combination of FK866 and 2-HNA achieved the lowest CI and the highest cytotoxicity in the cell viability studies.

In keeping with the cell viability experiments, the test compounds IM 29, IM 38, MMB-128, MMB-131, and MMB-268 were found to mimic the effect of 2-HNA on intracellular NAD^+^ levels and thus represent a set of potential NAPRT inhibitors. Indeed, co-treatments with the cited compounds at a 100 μM concentration and 30 nM FK866 produced a marked drop in the NAD^+^ levels (42.9–56.4%). A similar decrease in NAD^+^ was obtained in response to the NAMPT inhibitor combined with compound IM 49. Nevertheless, compound IM 49 by itself strongly reduced the OVCAR-5 intracellular NAD^+^ levels. Therefore, a lower degree of synergism was observed between this test compound and FK866. Possibly, IM 49 can interfere with other enzyme(s) that are involved in NAD synthesis in addition to hNAPRT.

Besides the test compounds that effectively cooperate with FK866 in decreasing NAD^+^ levels, the co-administration of compounds IM 43 and MMB-312 with FK866 only yielded additive effects in terms of NAD^+^ reduction in OVCAR-5 cells. As depicted in Figure 3, cotreatments with the cited compounds and FK866 did not cause a major reduction of the NAD^+^ levels. These results appear not to be in line with our cell viability assay, where IM 43 exerted high cytotoxicity in combination with FK866 in OVCAR-5 cells, while MMB-312 achieved the lowest CI value (CI = 0.12) out of all the tested compounds. These results suggest that other mechanisms may come into play to justify the synergy between IM 43 or MMB-312 and FK866, which need to be addressed in further studies.

### 2.3. Inhibition of the Recombinant hNAPRT

**Figure 4:**
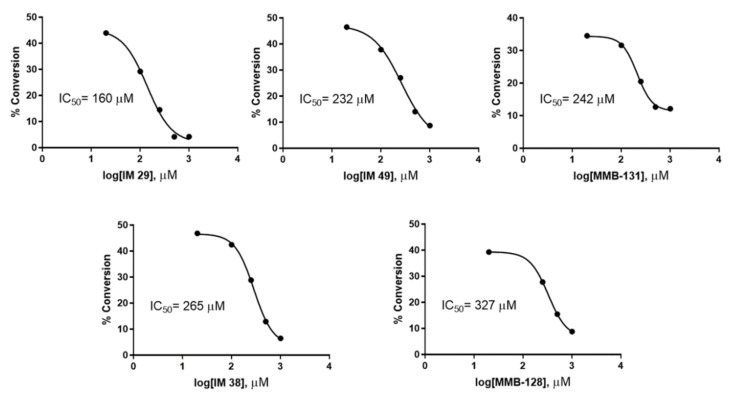
Compounds IM 29, IM 49, MMB-131, IM 38, and MMB-128 inhibit recombinant hNAPRT in the μM range. The test compounds were added at different concentrations to reaction mixtures containing hNAPRT and substrates and the half maximal inhibitory concentration (IC_50_) was obtained for each compound by measuring the amounts of NA and NAMN that was present after the reactions.

The six test compounds that were found to exert a synergistic effect with FK866 on OVCAR-5 intracellular NAD^+^ levels were tested in a biochemical assay with recombinant hNAPRT as inhibitors of the enzyme catalytic activity. These inhibition studies resulted in the identification of five new hNAPRT inhibitors with IC_50_ values in the micromolar range. Compound IM 29 exhibited the highest activity against the formation of nicotinic acid mononucleotide with an IC_50_ of 160 μM (Figure 4). Comparable results were obtained for compounds IM 38, IM 49, MMB-128, and MMB-131, with estimated IC_50_ in the 200–300 μM range. This enzymatic assay confirmed that the observed cooperation between FK866 and the tested compounds in decreasing OVCAR-5 cell viability and intracellular NAD^+^ levels was due to the ability of the compounds to inhibit hNAPRT. Noteworthy, compound MMB-268 showed negligible inhibition of hNAPRT (data not shown).

### 2.4. hNAPRT Melting Temperature Experiment

**Figure 5:**
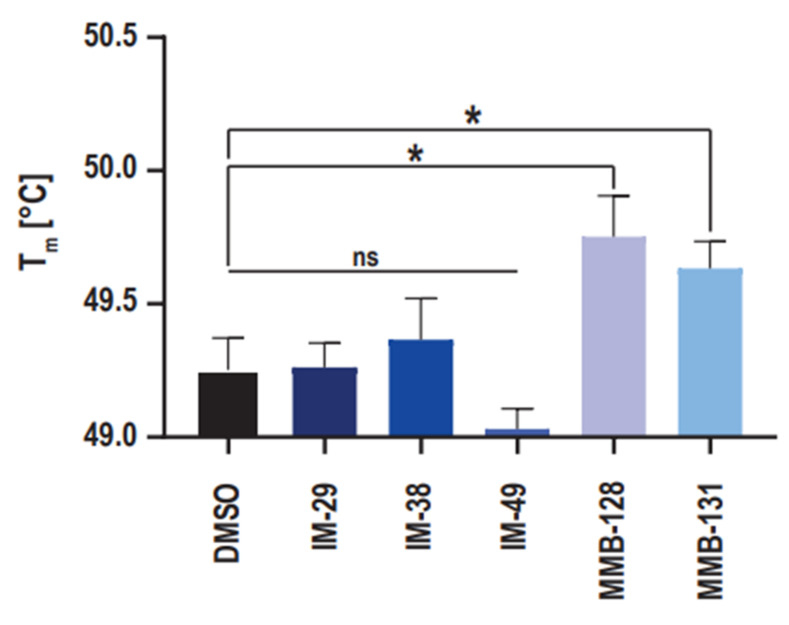
NAPRT inhibitors MMB-128 and MMB-131 directly engage recombinant hNAPRT. Differential scanning fluorimetry (DSF) was used to determine the thermal stabilization of hNAPRT protein upon inhibitor binding. Recombinant hNAPRT protein was exposed to 100 μM hNAPRT inhibitors over a defined temperature gradient and the melting temperature Tm was calculated for each compound. The data are shown as means ± standard error of the means (SEM) of two technical replicates with three biological replicates each. * *p* < 0.05; unpaired *t*-test.

With the purpose to provide support to the results that were obtained in the enzymatic activity assay, the ability of the new hNAPRT inhibitors to stabilize the protein through binding was evaluated via the measurement of the hNAPRT melting temperature in the presence or absence of the inhibitors at a 100 μM concentration. The resulting Tm shifts are depicted in Figure 5. Interestingly, a significant positive shift by approximately 0.5 °C in the Tm of hNAPRT was induced by compounds MMB-128 and MMB-131, suggesting a certain affinity of the inhibitors towards the protein that results in thermal stabilization. On the contrary, the melting temperature of hNAPRT remained unchanged when the enzyme was exposed to compounds IM 29, IM 38, or IM 49. A plausible explanation for this outcome is that larger compounds are more likely to establish a higher number of interactions and thus result in greater stabilization of a given protein. Indeed, significant Tm shifts were reached with the two largest inhibitors (MMB-128 and MMB-131).

### 2.5. Analysis of Inhibitor Binding Pose

**Figure 6:**
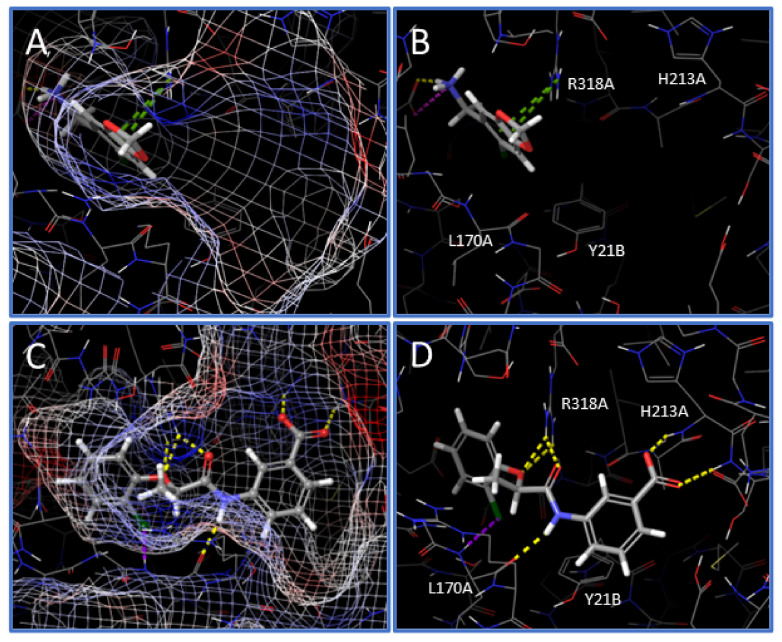
Binding mode that is predicted by molecular docking of compounds IM 29 (**A**,**B**) and IM 38 (**C**,**D**) in the hNAPRT active site. The analysis of the docking binding poses was performed on the academic version of Schrodinger Maestro v.2017-4. Protein is represented in thin sticks whereas ligands are depicted in thick tubes. Hydrogen bonds appear as yellow dotted lines. Salt bridges are represented by pink dotted lines. Pi-cation interactions are depicted as green dotted lines and halogen bonds are shown as purple dotted lines.

Following the in vitro characterization of the new hNAPRT inhibitors, a molecular docking on the hNAPRT structure was performed to shed light on the binding mode features that are likely related to the enzyme inhibition. Docking of compound IM 29 shows that the small and rigid molecule binds deep into the active site pocket of hNAPRT and it is stabilized by several interactions with protein residues (Figure 6A,B). Remarkably, IM 29 establishes two pi-cation contacts with ARG318A, a residue that is reported to exert a key role in catalysis that is potentially hampered by IM 29 binding [28,29]. The ionization at a physiological pH of the amino group of IM 29 appears relevant for engagement and inhibition of hNAPRT. Indeed, the carboxylate side chain of GLU167A in proximity to the ammonium moiety of IM 29 indicates suitable ligand-receptor electrostatic complementarity and results in a salt bridge and short-distance hydrogen bond between the cited amino acid and the hNAPRT inhibitor.

As for compound IM 29, docking into the hNAPRT active site provides a favorable binding mode for IM 38. The ligand fits in the active site maintaining the inherent planarity of its amide conjugated system, resulting in negligible ligand strain. Electrostatic potential mapping of the protein (Figure 6C) shows that the most electronegative atoms of the inhibitor are surrounded by positively charged active site residues. As for many drug candidates in medicinal chemistry, the affinity of hNAPRT towards IM 38 is largely explainable by the occurrence of numerous hydrogen bonds upon ligand binding. The carboxylate moiety of the ligand is key for its bioactivity, participating in two H-bonds with the backbone of hNAPRT residues HIS213A and SER214A (Figure 6D). Furthermore, through its amide and ether groups, IM 38 establishes several hydrogen bonds with the amino acids ARG318A and LEU170A. The latter residue also interacts with the chlorine atom of IM 38, albeit the poor directionality of the halogen bond suggests this interaction to be of limited relevance.

## 3. Discussion

Since NAD depletion emerged as a promising anticancer strategy, most of the research has focused on disabling the nicotinamide salvage pathway, which overall represents the main route to NAD biosynthesis in mammals. Indeed, several families of potent NAMPT inhibitors have emerged over the last decades with encouraging preclinical antitumor efficacy. Unfortunately, the expectations on some of these NAMPT inhibitors were not met later on in clinical trials, which showed limited clinical activity for these compounds. Piacente et al. hypothesized that alternative NAD production routes could represent a mechanism of tumor resistance to NAMPT inhibitors and demonstrated that the Preiss–Handler pathway gene, NAPRT, is frequently amplified and overexpressed in a subset of human tumors such as ovarian, breast, pancreatic, and prostate cancer. In the cited study, targeting NAPRT through silencing or chemical inhibition effectively sensitized NAPRT-expressing cancer cells to FK866 both in vitro and in vivo. In addition, the authors highlighted the need to discover new NAPRT inhibitors with increased potency with respect to 2-hydroxynicotinic acid and other active compounds that date back to the last century.

Here we report the first study that is aimed at identifying new hNAPRT inhibitors through a structure-based drug design approach with the evaluation of bioactivity with state-of-the-art in vitro assays.

As we were particularly interested in identifying active compounds in NAPRT-expressing cells, our first assay was designed as an in vitro screening to study which test compounds successfully sensitized OVCAR-5 cells to the NAMPT inhibitor FK866, essentially recreating the results that were obtained with the reference NAPRT inhibitor, 2-HNA. A total of eight compounds demonstrated synergism along with FK866 in decreasing cell viability, suggesting an impairment of the Preiss–Handler pathway. Most of these hits also showed the ability to cooperate with FK866 synergistically to decrease intracellular NAD^+^ levels in OVCAR-5 cells. Specifically, such an effect on intracellular NAD^+^ was observed in response to six compounds (IM 29, IM 38, IM 49, MMB-128, MMB-131, and MMB-268) when these were combined with FK866. Conversely, two compounds (IM 43 and MMB-312) showed promising results in the cell viability assay but failed to cooperate with FK866 to lower the NAD^+^ levels, suggesting that their antitumor effect may reflect an off-target activity.

Following cell assays, we proceeded to elucidate the regulatory properties of the best compounds on recombinant hNAPRT. A total of five compounds (IM 29, IM 38, IM 49, MMB-128, and MMB-131) showed inhibitory properties against hNAPRT and IC_50_ in the micromolar range. The suitability of our assays is supported by the fact that only one compound (MMB-268) out of the pool of candidates that exerted cytotoxicity and intracellular NAD^+^ decrease in combination with FK866 failed to inhibit hNAPRT enzymatic activity. Therefore, MMB-268 that showed promising activities on the OVCAR-5 cell line may be related to a biological target other than hNAPRT. For instance, one possibility is that MMB-268 could impair NAD synthetase (NADSYN) activity, catalyzing the conversion of NAAD into NAD independently of the Nam salvage biosynthetic pathway. NADSYN inhibition could lead to results that are comparable to hNAPRT inhibition in our in vitro cell assays. Finally, we decided to study the capability of the hNAPRT inhibitors that were herein identified to confer thermal stability to hNAPRT by DSF. Direct engagement of compounds MMB-128 and MMB-131 to hNAPRT was confirmed by positive shifts in the protein melting temperature when exposed to the inhibitors.

In summary, our findings broaden the chemical space of known hNAPRT inhibitors and pave the way for the identification of new NAD-lowering anticancer drugs. The new hNAPRT inhibitors that we report are characterized by a low molecular weight and, thus, are susceptible to undergo optimization studies shortly to increase their potency through chemical structure modifications.

## 4. Materials and Methods

### 4.1. High Throughput Virtual Screening

A high-throughput molecular docking screening was performed to discover new inhibitors of hNAPRT. The crystal structure of hNAPRT (PDB code: 4YUB) was retrieved from The Protein Data Bank [45]. This structure was prepared with the academic version of Schrodinger Maestro v.2017-4 with standard preparation procedures, that include the removal of water molecules, correct assignment of bond orders, the addition of hydrogens, and the optimization of protonation states and restrained energy minimization. A 15 Å grid centered on the enzymatically relevant active site residues LEU170A, ARG318A, and TYR21B was generated and docking virtual screening was run with the modeling tools that are offered by the Mcule platform, performing ligand preparation with Gypsum-DL using the default settings [28,29,46,47].

The docking results were thoroughly visually inspected considering widely accepted aspects in the scientific community such as quality of ligand-receptor interactions, docking score, fitting in the active site, ligand strain, and drug-likeness [48]. The most promising compounds were purchased or provided by collaborators and tested in vitro.

### 4.2. Virtual Screening Libraries

The chemical compounds that were subjected to docking screening were obtained from several sources. The list of libraries and providers is numbered below.

(1) Approximately 1 × 10^5^ molecules from the “Potentially purchasable compounds” Mcule database, selected based on simple physicochemical properties.
(2) The Prestwick Chemical Library^®^ [49].
(3) Approximately 2000 in-stock compounds from EDASA Scientific [50].
(4) A small selection of approximately 100 molecules that were synthesized by the University of Seville, some of which have already been described in the literature (see Supplementary Information for synthetic procedures and compounds characterization) [51,52,53,54].

Compounds were obtained in mg quantity and subsequently dissolved in DMSO to prepare a 50 mM stock solution. The purity of all compounds was > 95%, as declared by vendors or collaborators.

### 4.3. Cell Lines and Reagents

OVCAR-5 cells were obtained from the NCI-60 panel in 2015 as a kind gift from Prof. Zoppoli. The cells were passaged for less than six months before resuscitation for the experiments. Testing for mycoplasma was routinely done with the MycoAlert Mycoplasma Detection Kit (Lonza Group, Basel, Switzerland). The cells were maintained and treated with RPMI 1640 medium that was supplemented with 10% heat-inactivated FBS, penicillin (50 units/mL), and streptomycin (50 μg/mL) (Life Technologies, Monza, Italy). FK866 was kindly provided by the NIMH Chemical Synthesis and Drug Supply Program. 2-hydroxynicotinic acid was purchased (Sigma Aldrich S.r.l., Milan, Italy).

### 4.4. Cell Viability Assay

A total of 3 × 10^3^ OVCAR-5 cells/well were plated in 96-well plates in the regular culture medium. 24 h later the cell medium was removed and the cells were subsequently incubated either in the regular medium that was supplemented with 0.3 µM nicotinic acid (control wells) or in the treatment medium which contained 0.3 µM nicotinic acid and combinations of 100 nM FK866, 1 mM 2-HNA, and 100 µM of test compounds. Each condition was performed in triplicate and the cells remained under treatment for a total of 72 h at 37 °C. Thereafter, the culture plates were fixed with 10% trichloroacetic acid at 4 °C for 20 min, washed with cold water, and dried overnight. The plates were stained with 0.04% sulforhodamine B (SRB) in 1% acetic acid, washed four times with 1% acetic acid to remove the unbound dye, and dried overnight. Lastly, Trizma^®^-base 10 mM was added to the plates and cell viability was quantified by absorbance measurements on a Tecan Infinite^®^ 200 PRO instrument.

To quantify the extent of the interaction between FK866 and the putative NAPRT inhibitors or 2-HNA, we applied the combination index (CI) equation that is depicted below:

(1) $$\mathrm{CI}=\frac{mortality\;\%\;A+mortality\;\%\;B\;}{mortality\;\%\;\left(A+B\right)}$$

where: ‘*A*’ refers to the treatment of cells with FK866 100 nM; ‘*B*’ refers to the treatment of cells with the test compound 100 µM or 2-HNA 1 mM; ‘*A* + *B*’ refers to the coadministration of FK866 100 nM and the test compound at 100 µM or 2-HNA 1 mM. A synergistic interaction is evidenced by CI < 1, additive effect produces CI close to 1, whereas CI > 1 corresponds to antagonistic effect.

### 4.5. Intracellular NAD Quantification

A total of 1 × 10^5^ OVCAR-5 cells/well were plated in 24-well plates in the regular culture medium. 24 h later the cell medium was removed and the cells were cultured either in the regular medium that was supplemented with 0.3 µM nicotinic acid (control wells) or in the treatment medium (regular medium with 0.3 µM nicotinic acid and combinations of 30 nM FK866, 1 mM 2-HNA and 100 µM of test compounds). Each condition was performed in duplicate and the cells remained under treatment for a total of 20 h at 37 °C. Thereafter, the cell medium was removed and the cells were harvested and lysed with 0.6 M perchloric acid (PCA). Samples in PCA were neutralized by diluting the extracts in 100 mM sodium phosphate buffer (pH 8) and the total intracellular NAD^+^ content was determined with a sensitive enzyme cycling assay that exploits the use of alcohol dehydrogenase [55]. The obtained NAD^+^ values were normalized to cell lysate protein content that was quantified by the Bradford method.

### 4.6. Recombinant hNAPRT Production

The production of recombinant hNAPRT employed *E. coli* bacteria as an expression system. The human coding sequence for NAPRT was codon optimized for the expression in *E. coli* and cloned in a pET-23a vector containing a C-terminal His-tag by GenScript. A total of 5 ml of bacterial culture was grown overnight at 37 °C in the Luria–Bertani medium that was supplemented with 100 µg/mL ampicillin. The day after the culture was diluted at 1:100 in fresh medium and incubated at 25 °C. When a 0.3–0.4 OD_600_ was reached, protein expression was started at 20 °C by adding 1 mM IPTG, followed by overnight incubation.

The induced cells were harvested by mild centrifugation (5000 rpm, 10 min) in a Beckman Coulter J6-HC centrifuge with JA-10 rotor and resuspended in 1:50 original volume with equilibrium buffer (100 mM K_2_HPO_4_, 300 mM KCl, and 5 mM imidazole, pH 7.4). After sonication (18 × 10 s), the crude extract was clarified by centrifugation (6000 rpm, 15 min) with JA-20 rotor, and purified by His-tag affinity chromatography as follows. The supernatant was batch-mixed (1 h) with a HisPur Cobalt resin (Thermo Fisher Scientific, Pittsburg, PA, USA), previously equilibrated in the above equilibrium buffer, and then packed into a chromatographic column. The flow-through and the subsequent 10 mM imidazole wash buffer were discarded. The recombinant protein was eluted by equilibration buffer containing 150 mM imidazole.

After centrifuge-assisted protein concentration in a Protein Concentrator 10K (Pierce-Thermo Fisher Scientific, Pittsburg, PA, USA), the hNAPRT amount was quantified by absorbance measurement at 280 nm. The solution containing the protein was dialyzed overnight against 50 mM Tris/HCl, pH 7.4, 10 mM KCl, and 1 mM DTT, to remove imidazole and change the buffer with the reaction buffer. To improve enzyme stability, after dialysis, 0.5 mM PRPP and 20% glycerol were added and the protein was aliquoted and kept at −20 °C. All the steps were performed at 4 °C.

### 4.7. hNAPRT Inhibition Assay

hNAPRT was expressed in *E. coli* with an *N*-terminal His-tag and purified as described above. The enzymatic reactions were carried out at 37 °C in standard reaction mixtures containing 50 mM Tris-HCl, pH 7.4, 10 mM KCl, 2 mM MgCl_2_, 100 µM nicotinic acid, 200 µM PRPP, 0.1 mg/mL purified recombinant hNAPRT, and the test compound at five different concentrations ranging from 20 µM to 1000 µM.

Blank mixtures without compounds but with equal amounts of DMSO were set in parallel and their rates fixed as 100% activity. NAPRT was pre-incubated with the compounds for 5 min and reactions were started by the addition of the enzyme substrates and stopped after 45 min of incubation by heating to 85 °C for 3 min. Following centrifugation of the reaction mix, the supernatant was analyzed by HPLC by injection into a reverse-phase column (XTerra MS C18 Column, 125Å, 5 μm, 4.6 × 150 mm, Waters). The eluted species were monitored at 260 nm and the peaks of nicotinic acid and nicotinic acid mononucleotide were quantified with reference to standard curves. The percentage of nicotinic acid conversion was calculated for each reaction and the IC_50_ values for the active compounds were determined with GraphPad Prism 8 software (GraphPad Software, S. Diego, CA, USA).

### 4.8. NAPRT Melting Temperature Determination

The melting temperature ™ of hNAPRT was determined through the differential scanning fluorimetry (DSF) technique. Triplicates of 100 μM of each test compound and DMSO were added to a 96-well clear bottom BioRad PCR plate. Subsequently, DSF protein buffer, containing 5 μM of recombinant hNAPRT protein and 5X SPYRO^®^ Orange (S5692, Sigma-Aldrich) in 20 mM HEPES pH 7.5, 100 mM NaCl, 10 mM Mg-acetate, and 1 mM DTT was added. The plate was sealed and exposed to a temperature gradient from 20 to 95 °C in a BioRad CFX96 Real-Time System. The fluorescence for each temperature increment was measured at 465–580 nm with an excitation wavelength of 465 nm. The DSF templates that were provided by Niesen et al. were used for data analysis followed by GraphPad Prism 8 for statistical analysis of the generated data [56].

### 4.9. Statistical Analyses

All the experiments were repeated at least 3 times. Statistics were performed with GraphPad Prism v.8 software (GraphPad Software, S. Diego, CA, USA). All the parameters were tested by paired *t*-test or one-way ANOVA followed by the Tukey test. *p*-values < 0.05 were considered significant.

## Figures and Tables

**Table 1:** The number of in silico screening hit compounds that were obtained from each provider after visual inspection of the top-score docking binding poses.

Provider	Number of Compounds
University of Seville	22
MCULE	16
EDASA	13
Prestwick	11

## Data Availability

Not applicable.

## Supplementary Materials

The following supporting information can be downloaded at: https://www.mdpi.com/article/10.3390/ph15070855/s1, Table S1: Details of chemical structures of tested compounds as putative hNAPRT inhibitors. Figure S1: Nicotinic acid in culture medium determines the susceptibility of the OVCAR-5 cell line to treatments with FK866 and 2-hydroxynicotinic acid.
