# Supplementary material for: Structure-Based Identification and Biological Characterization of New NAPRT Inhibitors
Source: Pharmaceuticals (Basel). 2022 Jul 12;15(7):855. doi: 10.3390/ph15070855 (PMC9320560; doi:10.3390/ph15070855)
Supplement: Supplementary file 1 [file pharmaceuticals-15-00855-s001.zip › pharmaceuticals-1799864-supplementary.pdf]

Supplementary information

COMPOUND SYNTHESIS

(2*S*,3*S*,4*R*)-2-[2-(4-Phenyl-1*H*-1,2,3-triazol-1-yl)acetylaminomethyl]-pyrrolidine-3,4-diol (MMB-107)

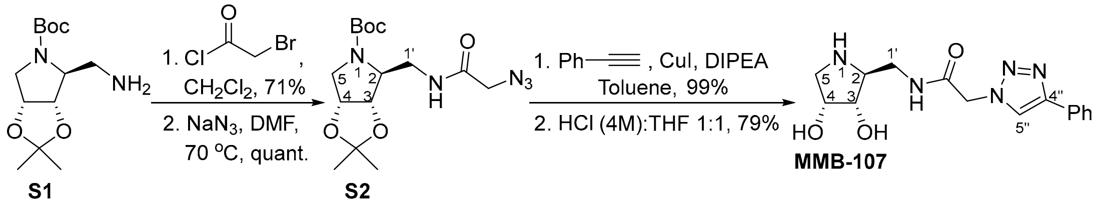

To a solution of **S1** [Ref.A] (245 mg, 0.899 mmol) in anhydrous CH_2Cl_2 (9 mL) at 0 °C, bromoacetyl chloride (283 mg, 1.80 mmol) and Et_3N (256 μL, 1.80 mmol) were added and the mixture was stirred at r.t. for 2 h. The solution was washed with sat. aq. soln. of $NaHCO_3$, water and brine and the organic layer was dried over Na_2SO_4, filtered and evaporated. The resulting residue was purified by chromatography column on silica gel (EtOAc:cyclohexane 1:1) to give the corresponding protected pyrrolidine-(bromo)amide (252 mg, 0.641 mmol, 71%) as an oil. To a solution of this compound (234 mg, 0.595 mmol) in DMF (5 mL), NaN_3 (97 mg, 1.5 mmol) was added. After stirring at 70 °C for 2 h, the solvent was removed under reduced pressure and the crude product was dissolved in CH_2Cl_2, washed with water and brine, dried over Na_2SO_4, filtered and evaporated. The resulting residue was purified by chromatography column on silica gel (EtOAc:cyclohexane 1:1) to give **S2** (213 mg, 0.595 mmol, quantitative) as a colourless oil.

Characterizacion data for S2. $[\alpha]_D^{26}$ + 31.1 (*c* 0.79, CH_2Cl_2). IR (ν cm^{-1}) 3304 (NH), 2980, 2105 (N_3), 1668 (C=O), 1537, 1407, 1366, 1158, 1052, 853. ^{1}H-NMR (300 MHz, $CDCl_3$, δ ppm, *J* Hz, mixture of rotamers) δ 7.25, 6.58 (br. s, 1H, NH), 4.70 (ap. t, 1H, H-4), 4.45-4.43 (m, 1H, H-3), 4.27-4.10 (m, 1H, H-2), 4.02-3.82 (m, 3H, -C*H*$_2$N$_3$, H-5a), 3.47-3.22 (m, 3H, H-1′a, H-5b, H-1′b), 1.47 (s, 9H, -C(C*H*$_3$)$_3$), 1.43 (s, 3H, -C(C*H*$_3$)$_2$), 1.29 (s, 3H, -C(C*H*$_3$)$_2$). ^{13}C-NMR (75.4 MHz, $CDCl_3$, δ ppm, mixture of rotamers) δ 167.5 (C=O, amide), 155.7 (C=O, Boc), 112.2 (-*C*(CH$_3$)$_2$), 83.2, 82.6 (C-3), 80.8 (-*C*(CH$_3$)$_3$), 79.4, 78.9 (C-4), 63.5, 62.6 (C-2), 52.8 (-*C*H$_2$N$_3$), 51.5, 51.1 (C-5), 41.3, 40.0 (C-1′), 28.4 (-C(*C*H$_3$)$_3$), 27.0 (-C(*C*H$_3$)$_2$), 25.1 (-C(*C*H$_3$)$_2$). HRESIMS *m/z* found 378.1745, calc. for $C_{15}H_{25}N_5O_5Na$ $[M+Na]^+$: 378.1748.

To a solution of **S2** (143 mg, 0.402 mmol) in toluene (4 mL), phenylacetylene (91 μL, 0.81 mmol), DIPEA (268 μL, 1.53 mmol) and CuI (47 mg, 0.24 mmol) were added and the solution was stirred at r.t. for 2 h. The solvent was evaporated and the resulting residue was dissolved in EtOAc and sat. aq. soln. of $NaHCO_3$ was added. The aqueous phase was extracted with EtOAc (x2) and the organic layers were dried over Na_2SO_4, filtered and evaporated. The resulting residue was

purified by chromatography column on silica gel (EtOAc:cyclohexane 2:1→4:1) to give the corresponding protected pyrrolidine-triazole (182 mg, 0.398 mmol, 99%) as a colourless solid. A solution of this compound (87 mg, 0.19 mmol) in HCl (4M):THF (1:1, 4.6 mL), was stirred at r.t. for 3 h. Evaporation of the solvent and chromatographic purification on Dowex 50WX8 eluting with MeOH, H_2O and NH_4OH 25%, yielded **MMB-107** (49 mg, 0.15 mmol, 79%) as a white solid.

Characterizacion data for MMB-107. $[\alpha]_D^{26}$ – 26.0 (*c* 0.56, DMSO). IR (ν cm^{-1}) 3305, 3239 (OH, NH), 2902, 1662 (C=O), 1566, 1464, 1277, 1083, 835, 760. ^{1}H-NMR (300 MHz, DMSO-d_6, δ ppm, *J* Hz) δ 8.51 (s, 1H, H-5′′), 8.30 (t, 1H, $J_{NH,1'}$ = 5.3, -N*H*CO), 7.87-7.85 (m, 2H, H-aromat.), 7.48-7.43 (m, 2H, H-aromat.), 7.36-7.30 (m, 1H, H-aromat.), 5.15 (s, 2H, -CH_2CO), 4.53 (br. s, 2H, OH-3, OH-4), 3.86 (ap. q, 1H, H-4), 3.49 (ap. t, 1H, H-3), 3.39-3.24 (m, 2H, H-1′a, NH), 3.11-3.04 (m, 1H, H-1′b), 3.03-2.97 (m, 1H, H-5a), 2.95-2.89 (m, 1H, H-2), 2.64 (dd, 1H, $^2J_{5b,5a}$ = 11.1, $J_{5b,4}$ = 4.1, H-5b). ^{13}C-NMR (75.4 MHz, DMSO-d_6, δ ppm) δ 165.5 (C=O), 146.1 (C-4′′), 130.8 (Cq aromat.), 128.9 (C aromat.), 127.8 (C aromat.), 125.1 (C aromat.), 122.9 (C-5′′), 74.3 (C-3), 70.8 (C-4), 61.7 (C-2), 51.8 (-CH_2CO), 51.6 (C-5), 42.2 (C-1′). HRESIMS *m*/*z* found 318.1559, calc. for $C_{15}H_{20}N_5O_3$ $[M+H]^+$: 318.1561.

(2*R*,3*R*,4*S*)-2-Benzoylaminomethyl-pyrrolidine-3,4-diol (MMB-89)

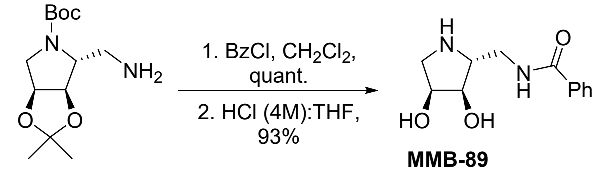

To a solution of ***ent*-S1** [Ref.D] (67 mg, 0.25 mmol) in anhydrous CH_2Cl_2 (3 mL) at 0 °C, benzoyl chloride (58 mL, 0.50 mmol) and Et_3N (70 mL, 0.49 mmol) were added and the mixture was stirred at r.t. for 2 h. The solution was washed with sat. aq. soln. of $NaHCO_3$, water and brine and the organic layer was dried over Na_2SO_4, filtered and evaporated. The resulting residue was purified by chromatography column on silica gel (EtOAc:cyclohexane 1:2→1:1) to give the corresponding protected pyrrolidine-amide (95 mg, 0.25 mmol, quantitative) as a colourless oil. A solution of this compound (56 mg, 0.15 mmol) in HCl (4M):THF (1:1, 3.6 mL), was stirred at r.t. for 3 h. Evaporation of the solvent and chromatographic purification on Dowex 50WX8 eluting with MeOH, H_2O and NH_4OH 25%, yielded **MMB-89** (33 mg, 0.14 mmol, 93%) as a white solid.

Characterizacion data for MMB-89. $[\alpha]_D^{26}$ + 36.2 (*c* 0.74, MeOH). HRESIMS *m*/*z* found 237.1230, calc. for $C_{12}H_{17}N_2O_3$ $[M+H]^+$: 237.1234. IR and NMR data match with those of its enantiomer [Ref.A].

(2*R*,3*R*,4*S*)-2-[2-(4-Phenyl-1*H*-1,2,3-triazol-1-yl)acetylaminomethyl]-pyrrolidine-3,4-diol (MMB-109)

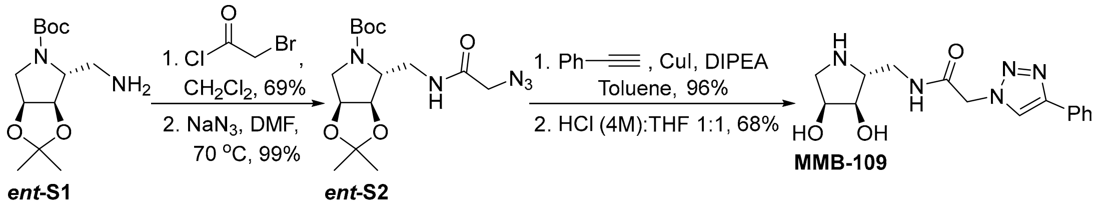

To a solution of ***ent*-S1** [Ref. D] (198 mg, 0.727 mmol) in anhydrous CH_2Cl_2 (7 mL) at 0 °C, bromoacetyl chloride (229 mg, 1.46 mmol) and Et_3N (208 μL, 1.46 mmol) were added and the mixture was stirred at r.t. for 2 h. The solution was washed with sat. aq. soln. of $NaHCO_3$, water and brine and the organic layer was dried over Na_2SO_4, filtered and evaporated. The resulting residue was purified by chromatography column on silica gel (EtOAc:cyclohexane 1:1) to give the corresponding protected pyrrolidine-(bromo)amide (198 mg, 0.503 mmol, 69%) as an oil. To a solution of this compound (187 mg, 0.476 mmol) in DMF (4.5 mL), NaN_3 (78 mg, 1.2 mmol) was added. After stirring at 70 °C for 2 h the solvent was removed under reduced pressure and the obtained residue was dissolved in CH_2Cl_2, washed with water and brine, dried over Na_2SO_4, filtered and evaporated. The crude product was purified by chromatography column on silica gel (EtOAc:cyclohexane 1:1) to give ***ent*-S2** (167 mg, 0.470 mmol, 99%) as a colourless oil.

Characterizacion data for *ent*-S2. $[\alpha]_D^{26}$ – 32.3 (*c* 0.81, CH_2Cl_2). HRESIMS *m/z* found 378.1746, calc. for $C_{15}H_{25}N_5O_5Na$ $[M+Na]^+$: 378.1748. IR and NMR data match with those of compound **S2**.

To a solution of ***ent*-S2** (140 mg, 0.394 mmol) in toluene (3.5 mL), phenylacetylene (89 μL, 0.79 mmol), DIPEA (262 μL, 1.50 mmol) and CuI (46 mg, 0.24 mmol) were added and the solution was stirred at r.t. for 1.5 h. The solvent was evaporated and the resulting residue was dissolved in EtOAc and sat. aq. soln. of $NaHCO_3$ was added. The aqueous phase was extracted with EtOAc (x2) and the organic layers were dried over Na_2SO_4, filtered and evaporated. The resulting residue was purified by chromatography column on silica gel (EtOAc:cyclohexane 2:1→4:1) to give the corresponding protected pyrrolidine-triazole (173 mg, 0.378 mmol, 96%) as a colourless solid. A solution of this compound (86 mg, 0.19 mmol) in HCl (4M):THF (1:1, 4.6 mL), was stirred at r.t. for 3 h. Evaporation of the solvent and chromatographic purification on Dowex 50WX8 eluting with MeOH, H_2O and NH_4OH 25%, yielded **MMB-109** (42 mg, 0.13 mmol, 68%) as a white solid.

Characterizacion data for MMB-109. $[\alpha]_D^{26}$ + 29.3 (*c* 0.61, DMSO). HRESIMS *m/z* found 318.1560, calc. for $C_{15}H_{20}N_5O_3$ $[M+H]^+$: 318.1561. IR and NMR data match with those of compound **MMB-107**.

REFERENCES

Ref. A: Martínez-Bailén, M.; Carmona, A.T.; Moreno-Clavijo, E.; Robina, I.; Ide, D.; Kato, A.; Moreno-Vargas, A.-J. Tuning of β-glucosidase and α-galactosidase inhibition by generation and *in situ* screening of a library of pyrrolidine-triazole hybrid molecules. *European Journal of Medicinal Chemistry* **2017**, *138*, 532–542, doi:10.1016/j.ejmech.2017.06.055.

Ref. B: Martínez-Bailén, M.; Carmona, A.T.; Patterson-Orazem, A.C.; Lieberman, R.L.; Ide, D.; Kubo, M.; Kato, A.; Robina, I.; Moreno-Vargas, A.-J. Exploring substituent diversity on pyrrolidine-aryltriazole iminosugars: Structural basis of β-glucocerebrosidase inhibition. *Bioorganic Chemistry* **2019**, *86*, 652–664,doi:10.1016/j.bioorg.2019.02.025.

Ref. C: Martínez-Bailén, M.; Galbis, E.; Carmona, A.T.; de-Paz, M.-V.; Robina, I. Preparation of water-soluble glycopolymers derived from five-membered iminosugars. *European Polymer Journal* **2019**, *119*, 213–221, doi:10.1016/j.eurpolymj.2019.07.027.

Ref. D: Popowycz, F.; Gerber-Lemaire, S.; Demange, R.; Rodríguez-García, E.; Carmona-Asenjo, A. T.; Robina, I.; Vogel, P. Derivatives of (2*R*,3*R*,4*S*)-2-aminomethylpyrrolidine-3,4-diol are selective α-mannosidase inhibitors. *Bioorganic & Medicinal Chemistry Letters* **2001**, *11*, 2489–2493, doi:10.1016/S0960-894X(01)00477-2.

NMR SPECTRA

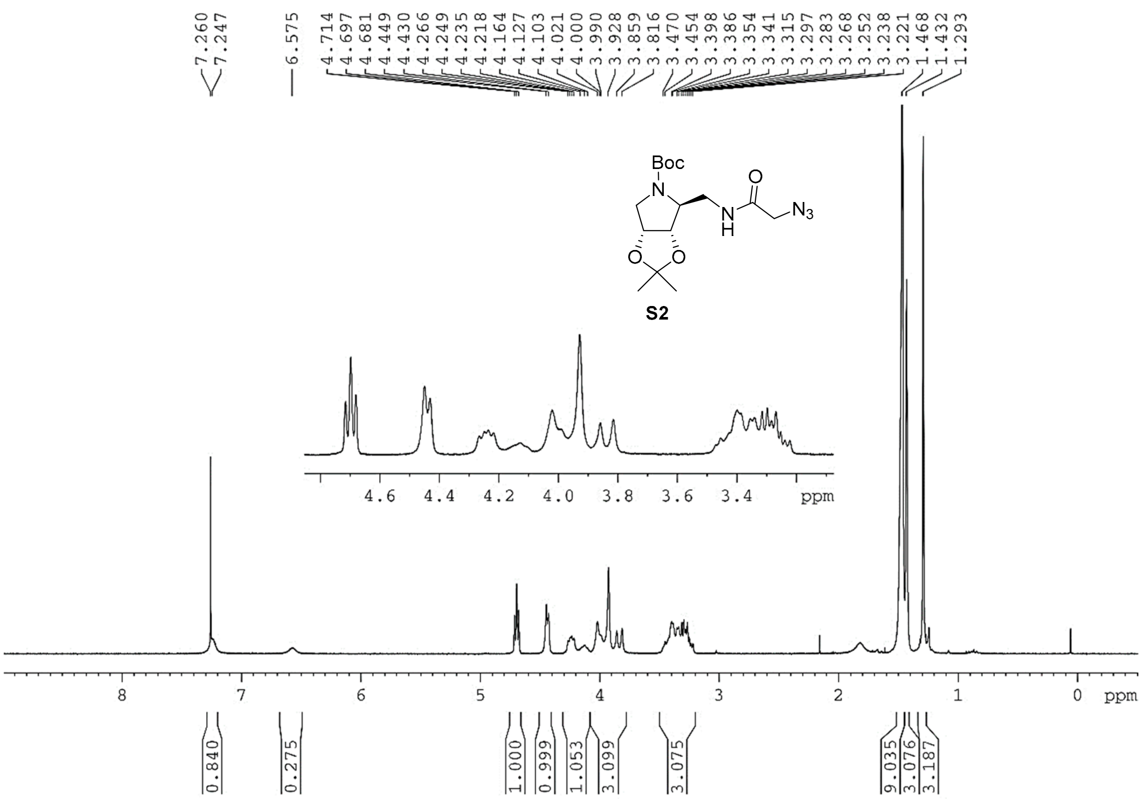

^{1}H-NMR (300 MHz, $CDCl_3$)

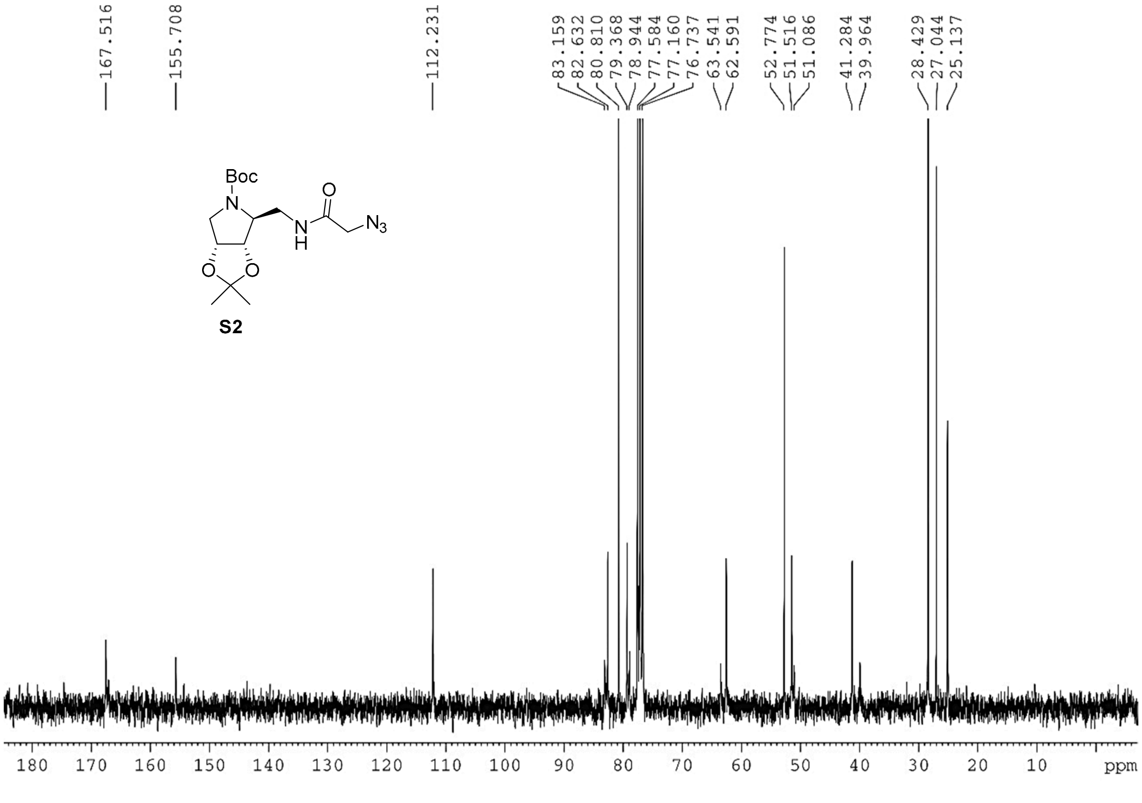

^{13}C-NMR (75.4 MHz, $CDCl_3$)

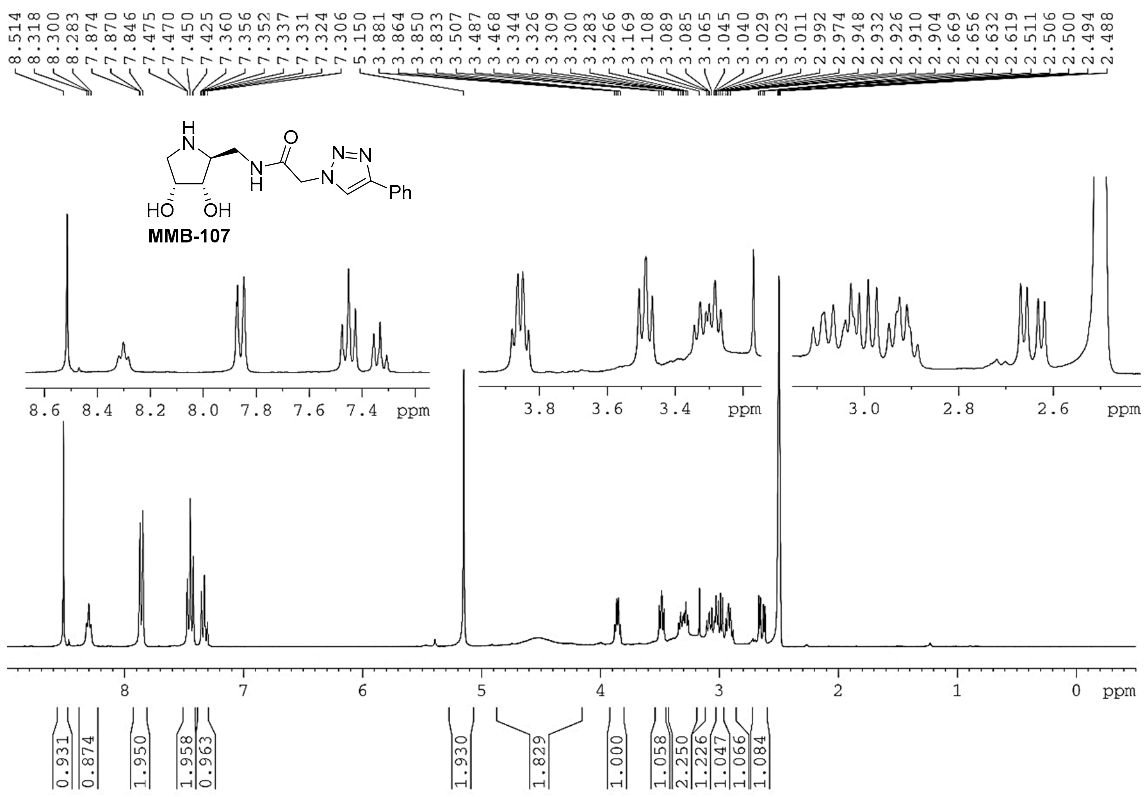

^{1}H-NMR (300 MHz, DMSO-d_6)

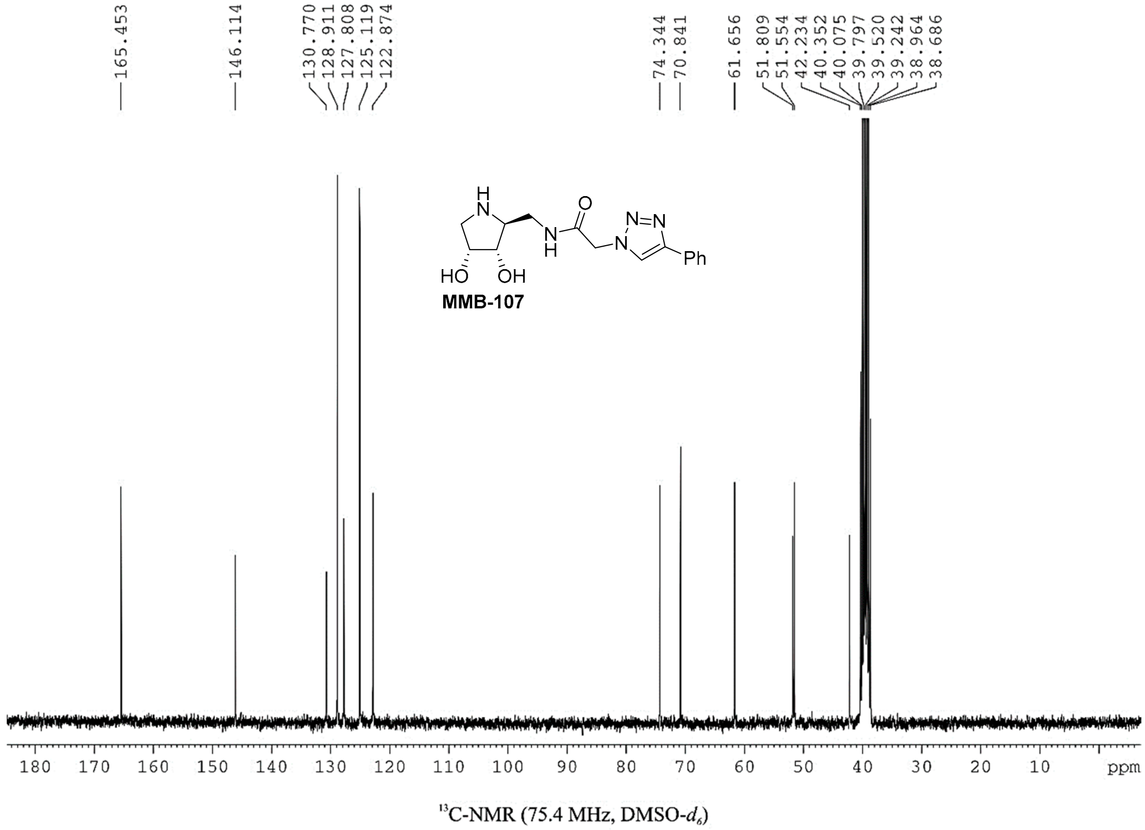

^{13}C-NMR (75.4 MHz, DMSO-d_6)

Table S1. Details of chemical structures of tested compounds as putative hNAPRT inhibitors.

Number	Provider	Compound ID	Structure
1	EDASA	IM 21	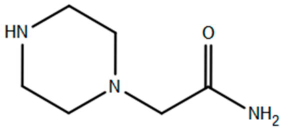
2	EDASA	IM 22	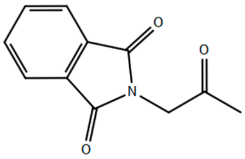
3	EDASA	IM 23	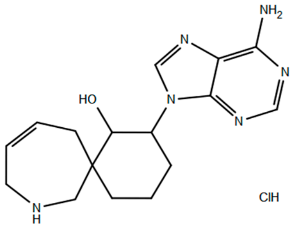
4	EDASA	IM 24	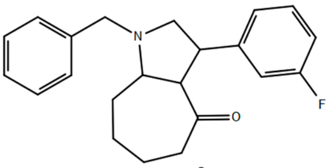
5	EDASA	IM 25	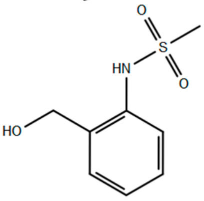
6	EDASA	IM 26	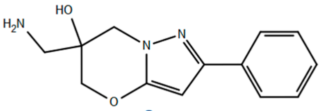
7	EDASA	IM 27	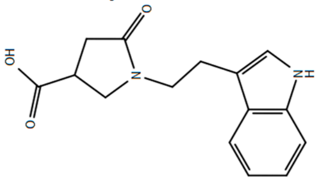
8	EDASA	IM 28	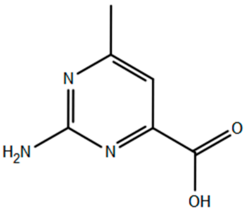
9	EDASA	IM 29	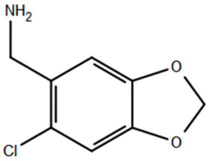

10	EDASA	IM 30	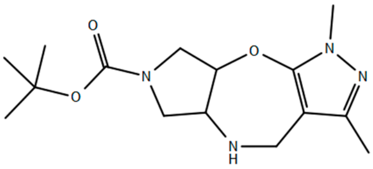
11	EDASA	IM 31	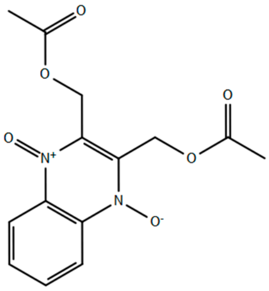
12	EDASA	IM 32	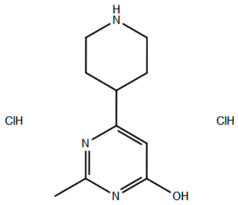
13	EDASA	IM 33	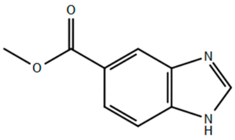
14	MCULE	IM 37	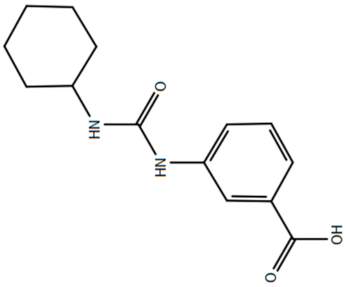
15	MCULE	IM 38	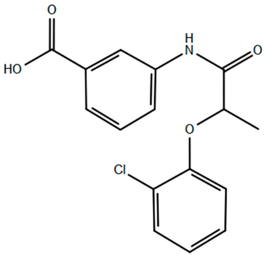
16	MCULE	IM 39	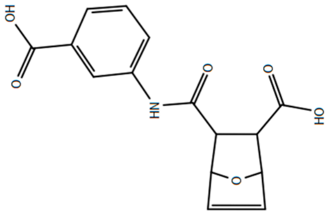
17	MCULE	IM 40	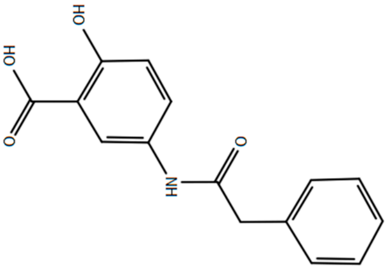

18	MCULE	IM 41	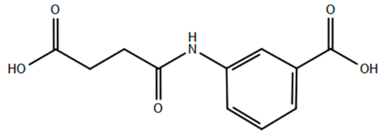
19	MCULE	IM 42	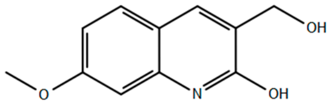
20	MCULE	IM 43	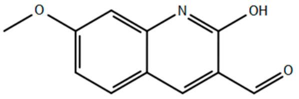
21	MCULE	IM 44	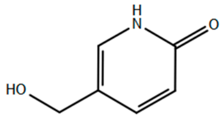
22	MCULE	IM 45	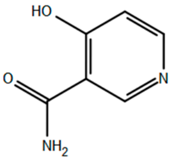
23	MCULE	IM 46	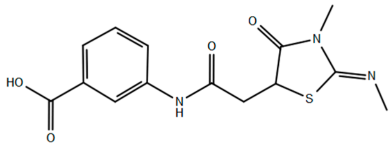
24	MCULE	IM 47	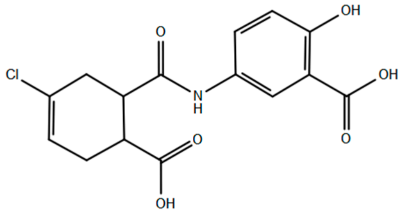
25	MCULE	IM 48	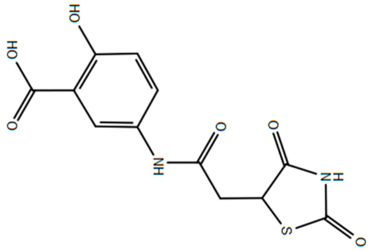
26	MCULE	IM 49	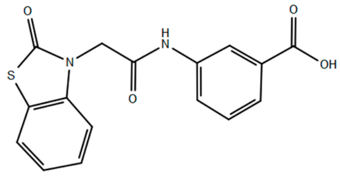
27	MCULE	IM 50	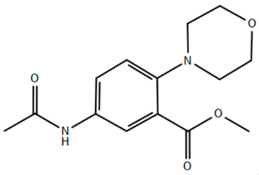
28	MCULE	IM 51	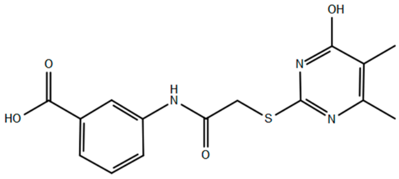

29	MCULE	IM 52	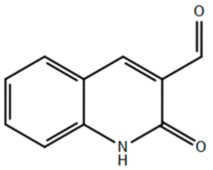
30	PRESTWICK	IM 53	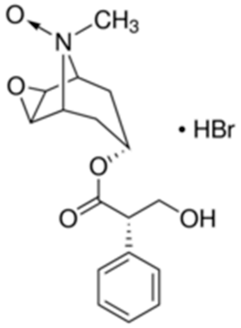
31	PRESTWICK	IM 54	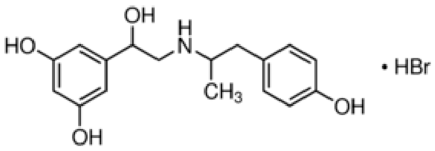
32	PRESTWICK	IM 55	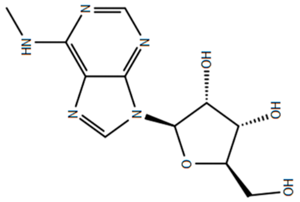
33	PRESTWICK	IM 56	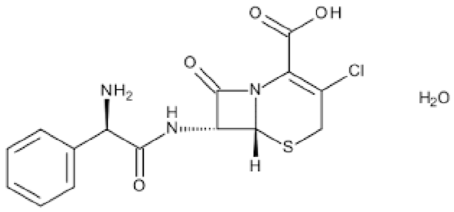
34	PRESTWICK	IM 57	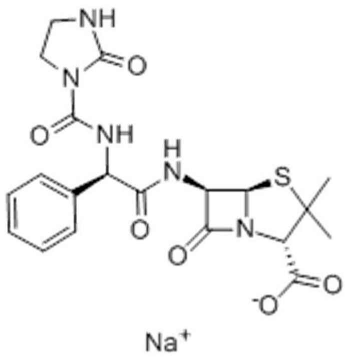
35	PRESTWICK	IM 58	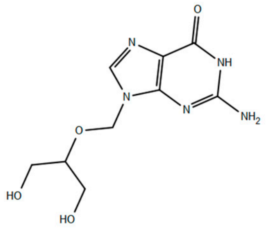
36	PRESTWICK	IM 59	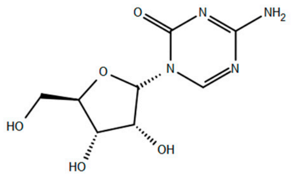

37	PRESTWICK	IM 60	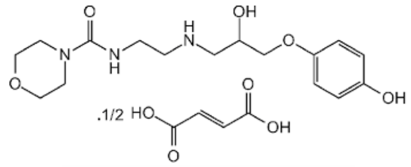
38	PRESTWICK	IM 61	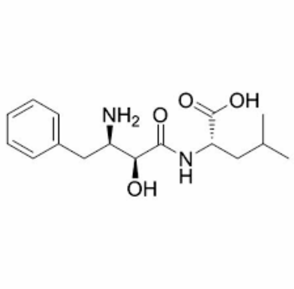
39	PRESTWICK	IM 62	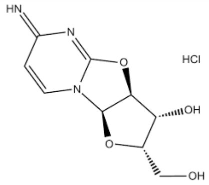
40	PRESTWICK	IM 63	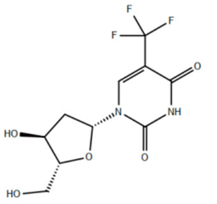
41	U. SEVILLE	MMB-69 [Ref. A]	
42	U. SEVILLE	MMB-105-B [Ref. A]	
43	U. SEVILLE	MMB-107	
44	U. SEVILLE	MMB-23-2 [Ref. A]	
45	U. SEVILLE	MMB-24 [Ref. A]	
46	U. SEVILLE	MMB-89	
47	U. SEVILLE	MMB-109	

48	U. SEVILLE	MMB-150 [Ref. A]	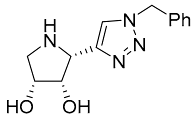
49	U. SEVILLE	MMB-238 [Ref. A]	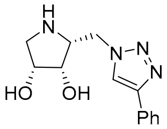
50	U. SEVILLE	MMB-239 [Ref. A]	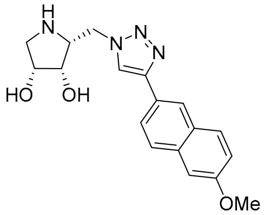
51	U. SEVILLE	MMB-241 [Ref. A]	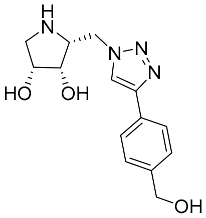
52	U. SEVILLE	CVL-16 [Ref. B]	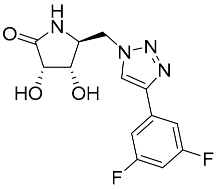
53	U. SEVILLE	MMB-131 [Ref. C]	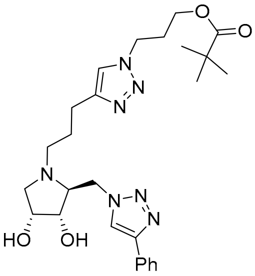
54	U. SEVILLE	MMB-128 [Ref. C]	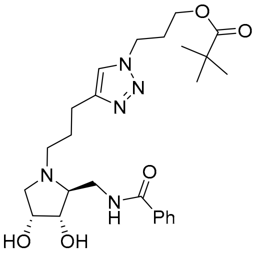
55	U. SEVILLE	MMB-294 [Ref. C]	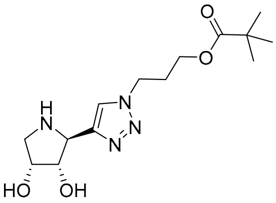
56	U. SEVILLE	MMB-136 [Ref. C]	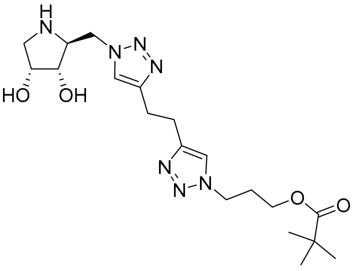

57	U. SEVILLE	MMB-266 [Ref. B]	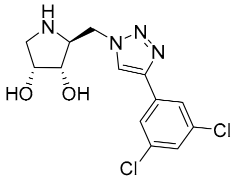
58	U. SEVILLE	MMB-310 [Ref. B]	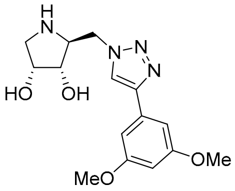
59	U. SEVILLE	MMB-260 [Ref. B]	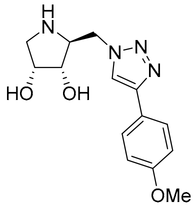
60	U. SEVILLE	MMB-311 [Ref. B]	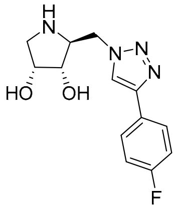
61	U. SEVILLE	MMB-268 [Ref. B]	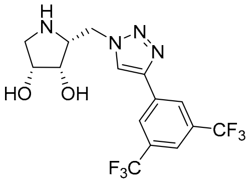
62	U. SEVILLE	MMB-312 [Ref. B]	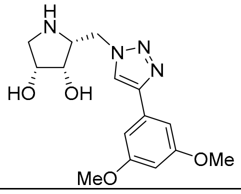

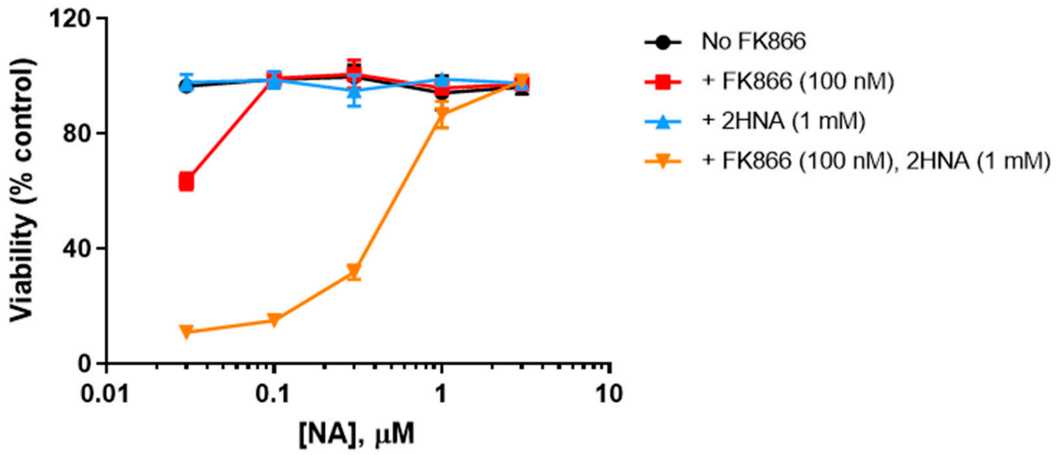

Figure S1. The concentration of nicotinic acid in culture medium determines the susceptibility of the OVCAR-5 cell line to treatments with FK866 and 2-hydroxynicotinic acid. Cells were cultured in RPMI 1640 medium supplemented with different concentrations of NA and combinations of FK866 and 2-hydroxynicotinic acid. Cell viability was determined following 72 h treatments and expressed as percentage of control cells.
